# Retrospective Study of 222 Dogs Suffering from Food-Responsive Enteropathy—Correlation with Clinical Variables, Diet and Breed

**DOI:** 10.3390/vetsci11070294

**Published:** 2024-07-01

**Authors:** Alessia Candellone, Gaia Raviri, Vittorio Saettone, Martine Didier, Giacomo Rossi, Andrea Marchegiani, Alessandra Gavazza, Alessandro Di Cerbo, Matteo Cerquetella

**Affiliations:** 1 NutriTO Vet srl, Via Bastone 4/2, 10095 Rosta, TO, Italy; 2 Endovet Group, 00118 Rome, Italy; 3 School of Biosciences and Veterinary Medicine, University of Camerino, Via Circonvallazione, 93/95, 62024 Matelica, MC, Italy; 4 White Bridge Pet Brands, Via Martin Piaggio 13/a, 16122 Genova, GE, Italy

**Keywords:** dog, food-responsive enteropathy, chronic inflammatory enteropathy, diet, breed, clinical progression

## Abstract

**Simple Summary:**

Food-responsive enteropathy is a chronic enteropathy of dogs managed with dietary changes; frequently, in such disease, more than one dietary trial is needed to resolve the condition. No clear predispositions are reported for the disease, and the response to the diet varies from subject to subject; additionally, very little literature is available about the possible role of the diet fed before the disease onset. The present study reports the clinical progression of 222 dogs diagnosed with food-responsive enteropathy (to the authors’ knowledge, the largest cohort of FRE dogs present in the international literature as single research) and compares clinical variables with diets and breeds. The authors believe that considering the number of patients included and the variables investigated, the present study could represent a reference for future ones.

**Abstract:**

Food-responsive enteropathy (FRE) is the most frequent form of canine chronic inflammatory enteropathy (CIE). It can be diagnosed if, after excluding known causes of diarrhea, clinical signs resolve or significantly improve after an appropriate dietary trial. No universal diet can resolve the clinical signs in every case of FRE, as genetic predisposition and environment (e.g., the possible role of the diet feed before the disease onset) are suggested as possible players. The study aimed to retrospectively evaluate the possible correlations between disease, diet, and breed in a large cohort of dogs (n = 222) suffering from FRE. Throughout the study, dogs differed based on dietary options: commercial diet group, homemade diet group, and mixed diet group. Diet, breed, age, body weight, body condition score (BCS), fecal score (FS), canine chronic enteropathy activity index (CCECAI), and selected clinical signs were variably evaluated at T0 and at final time (FT—based on response to the diet[s], but between 30 and 60 days). Significant differences between T0 and FT were found regarding FS, BCS, and CCECAI, as well as between age, BCS, and CCECAI at FT with the FS at FT. The CCECAI at FT was significantly directly correlated only with the shift from a mixed to a homemade diet. Finally, the multiple linear regression analysis between the covariables of different breeds versus clinical response to the dietary trials did not highlight any difference except for the passage from commercial to mixed diet in a specific subgroup of breeds. The present study reports the clinical progression in 222 dogs suffering from FRE, and it could represent a reference for the variables investigated, considering the large number of patients included.

## 1. Introduction

Food-responsive enteropathy (FRE) is a sub-group of canine chronic inflammatory enteropathies (CIEs). CIEs also include steroid-responsive (SRE) or immunosuppressant-responsive enteropathy (IRE) and non-responsive enteropathy (NRE); in the case of protein dispersion, such conditions can be further classified as protein-losing enteropathies (PLEs). The further existence of the so-called antibiotic-responsive enteropathy (ARE) is strongly debated [1,2]. Among CIEs, FRE is usually reported to be the most frequent one, variably representing around 2/3 of all cases of CIEs [2,3,4].

A CIE can be diagnosed as FRE if, after excluding known causes of diarrhea (e.g., fecal parasitosis or renal, liver, pancreatic insufficiency) per standard diagnostic protocols (e.g., fecal exams, CBC and blood chemistry, urinalysis, pancreatic and adrenal function, diagnostic imaging, etc.) [1,2], clinical signs resolve or significantly improve after an appropriate dietary trial, usually based on hydrolyzed protein commercial diet, novel (mono) protein commercial, or homemade diets [5,6,7]. These alimentary trials must be continued for at least 2–4 weeks, and in case of failure, at least a second attempt of the same duration must be made using a different diet [2]. It is important to underline that there is not a universal diet able to resolve the clinical signs in every case of FRE, as genetic predisposition and environment are suggested as key role players; furthermore, it is not always possible to certainly differentiate between cases of food allergy and food intolerance [4]. Regarding predisposing factors, very little literature is available about the possible role of the diet fed before the disease onset, but there is growing attention on this point, and, very interestingly, a recent study reports that dogs suffering from chronic enteropathy were less likely to be administered red meat as the primary protein source (vs. controls), while healthy controls were more likely to be fed a no-carbohydrate diet (vs. diseased dogs) [8].

Regarding genetics, several studies have demonstrated a greater incidence of FRE in certain dog breeds [9,10,11]. Reasons behind this predisposition, however, are still poorly understood, and different hypotheses have been postulated. Meyer et al. [12], for instance, have reported that large-breed dogs present an increased frequency of soft feces than small-breed ones when fed the same diet. This phenomenon may be related to the digestive peculiarities of large breed dogs compared to small ones (high intestinal and colonic permeabilities, prolonged colonic transit, and large caecum size), which exacerbate colonic fermentation and promote soft stool consistency [13]. Moreover, starch sources and forms play a crucial role in diet digestibility: purified carbohydrates are highly digestible for medium- and large-sized canines, while they are less suitable for miniature dogs, as they may promote constipation. For those latter patients, cereal flour is preferred [14]. It has been demonstrated that the domestication process may have contributed to canine adaptation to starch-rich diets. In particular, genetic selection has targeted a duplication affecting the *Amy2b* gene, which encodes for pancreatic amylase (the enzyme that breaks down starch into maltose in the small intestine), resulting in an average seven-fold *Amy2b* copy number expansion that is estimated to be associated with a 5.4% increase in serum amylase activity for each extra copy [15]. It was also revealed that canine domestication has altered pathways responsible for carbohydrate digestion and glucose absorption. Arendt et al. described a geographical pattern in the extent to which *Amy2b* copy numbers have expanded throughout the global canine population; they showed that dogs originating in regions where agriculture was practiced in prehistoric times carry significantly more *Amy2b* copies than dogs originating elsewhere [16]. The aforementioned genetic and metabolic peculiarities of dog breeds may influence the canine’s individual susceptibility to CIE and drive the clinical response to different elimination dietary trials.

The present study aimed to evaluate retrospectively the following in a large cohort of dogs suffering from FRE: (i) the possible correlation between disease/diet and breed/diet; (ii) the correlation among selected clinical variables during clinical remission/evolution; and (iii) the possible correlation between dietary management and clinical progression.

## 2. Materials and Methods

### 2.1. Patients

Client-owned dogs diagnosed with FRE in a two-year period (November 2021–November 2023) and referred to the NutriTO Vet group (a veterinary nutritional and gastroenterological consultancy referral service owned by one of the authors—AC), and to other referral facilities located in the Piemonte region (Italy) were retrospectively enrolled for the study. Written informed consent authorizing the use of clinical data for scientific purposes was obtained from all caregivers.

### 2.2. Inclusion/Exclusion Criteria

All dogs included in the study (T0) presented GI signs lasting at least three weeks, and all other possible extra-intestinal causes for their presence were excluded [1]. In particular, all dogs underwent complete blood count (CBC), serum biochemistry analysis, fecal flotation, and rapid test for *Giardia* spp. Other evaluations like serum cobalamin, folate, TLI, canine pancreatic specific lipase, adrenal function test (basal cortisol and ACTH stimulation test), complete urinalysis, abdominal radiographs and/or ultrasonographic examination, rectal cytology, and GI endoscopy were also variably performed, depending on the clinical presentation, in those cases in which they were deemed necessary to achieve a diagnosis. Patients who tested positive in fecal parasitological evaluations were excluded, except for a small sub-group of patients who tested positive for *Giardia* spp. but had already been treated with one or more cycles of standard therapy (fenbendazole) without resolving the clinical signs. These dogs were considered as suffering from giardiasis associated with dysbiosis, a condition in which the parasite is not necessarily responsible for the clinical presentation; such infection has even been suggested to protect children from moderate to severe diarrhea [17].

### 2.3. Dietary Management

Patients included in the study underwent one or more elimination diets for at least 30 to a maximum of 60 days (FT), depending on the clinical progression. Feed administered were one or more of the following: (i) commercial hydrolyzed diet, (ii) commercial mono-protein and mono-carbohydrate diet, (iii) commercial wet diet containing a single protein source and without carbohydrates, or (iv) homemade diet with foods that the animal had never eaten before. The first three options were included in the commercial diet group, while the fourth was in the homemade diet group; patients whose owners decided to administer both commercial and homemade diets were included in the mixed diet group. The diet determining the remission of GI signs had to be administered continuously for a further 4–6 weeks, without recording any relapse of clinical signs, to be considered effective in achieving the diagnosis. When choosing the dietary regime, the owners were free to choose one of the three options as the first- or second-line approach, and due to its retrospective nature, the present study about this aspect simply reports the results of this choice. Treats (if formulated with non-hydrolyzed proteins) were banned for the entire study period.

### 2.4. Parameters Investigated

As previously reported in the aim of the study, we recorded the breed of patients (divided into three subgroups based on the hypothetical expression of the *Amy2b* gene [16]) and the diet that patients were assuming at the time of inclusion (T0) (divided between three options: commercial, homemade, and mixed diet), as well as the diet resolving clinical signs at FT. Furthermore, two clinical scores were also monitored: the canine chronic enteropathy activity index (CCECAI) score (insignificant disease, 0–3; mild disease, 4–5; moderate disease, 6–8; severe disease, 9–11; very severe disease, >12 [18]), and the fecal score (FS) (from 1 = very hard and 2 = normal to 7 = watery diarrhea) [19]. Considering that some chronic patients, despite improving FS, continued to occasionally have poorly formed stools, to avoid a single episode of diarrhea yielding an altered perception of the real evolution of the clinical case, to assign the FS at FT, the average of the last 2–3 days was taken into account.

Additionally, other clinical variables/signs, in particular hematochezia/melena; constipation; vomiting/regurgitation/signs of gastro-esophageal reflux disorder (GERD); weight loss; anorexia/dysorexia; lethargy; dermatopathological signs (alopecia, pruritus, otitis); and physiological variables, particularly sex, age, body weight, and body condition score (BCS) (1–2–3 = too skinny; 4–5 = ideal weight; 6–7–8–9 = too fat) [20], both at T0 and FT (excluding sex and age), were also investigated.

### 2.5. Statistical Analysis

All data are expressed as mean ± SD and were analyzed using the Prism 9.0 software from GraphPad (San Diego, CA, USA). Differences in BCS, FS, CCECAI, and selected clinical variables/signs between T0 and FT were analyzed using a Wilcoxon matched-pairs signed rank test, while differences in body weight were analyzed using a paired *t*-test. Regarding correlations between age, weight, BCS, CCECAI, and FS, as well as between diet switch (commercial to homemade, commercial to mixed, homemade to commercial, homemade to mixed, mixed to commercial, and mixed to homemade) and CCECAI, a multiple linear regression analysis with a nonparametric Spearman’s correlation was applied. A *p* < 0.05 was considered significant.

## 3. Results

Two hundred twenty-two dogs were included in the study; 93 (42%) were females, and 129 (58%) were males, with a mean age of 5.2 ± 3.5 years (range: 6 months–12 years).

Regarding diet at T0, 142 dogs (63%) were fed a commercial diet, 31 (14%) a homemade one, and 49 (22%) a mixed diet; at FT, the number of patients/percentages referred to the three alimentary regimens passedrespectively to 56 (25%), 107 (48%), and 59 (27%).

Fifty-three different breeds (plus various mixed-breed dogs) were included in the study, and the individual numbers are reported in Table 1, divided based on the hypothetical expression of the *Amy2b* gene [16].

Values of body weight, BCS, FS, and CCECAI at T0 and FT are reported in Table 2.

Regarding the FS, 43 patients (19%) presented a normal value (2 or 3/7) at T0, while after the elimination diet, the number of dogs with normal FS increased to 163 (73%). The CCECAI at T0 was less than or equal to 3/27 in 62 (28%) dogs, while at FT, it was normal in 187 (84%) patients. In only 14 dogs, it was >6; the maximum was 12 in one dog. The frequency of the different clinical signs investigated is reported in Table 3.

The multiple linear regression analysis between the covariables considered (age, body weight, BCS, and CCECAI at FT with the FS at FT, as well as the various types of diet/dietary shift with the CCECAI at FT) highlighted the following main correlations (Table 4).

Among the first group of variables, BCS and FS were inversely related (*p* < 0.01), while CCECAI and FS were directly related (*p* < 0.001).

The CCECAI at FT was directly related only to the shift from a mixed to a homemade diet (*p* < 0.05).

Specifically, Table 5 shows the food choices made by the owners when defining the diet change as the first and possible second option.

Finally, the multiple linear regression analysis between the two covariables, different breeds versus clinical response to the dietary trials, did not highlight any difference, except for the passage from commercial to mixed diet in “red” breeds (*p* < 0.05). Bold represents total number of patients.

## 4. Discussion

The present study aimed to investigate possible genetic predispositions associated with specific diets and the clinical progression in a large cohort of dogs suffering from FRE. In a search of the international PubMed^®^ free research service for biomedical scientific literature (keywords: “food responsive enteropathy dog” and “food responsive diarrhea dog”), the results showed the present study, including 222 diseased dogs, this to be the one with the largest cohort of FRE dogs actually present in the international literature as a single study [21], and the authors believe it could represent a useful reference for this disease.

Directly connected the above, among the results of the present study, it is interesting to note that the mean age of patients included was 5.2 years. Indeed, previous authors reported that dogs affected by FRE are typically young and younger compared to dogs with other subclasses of CIEs [2,8,22]. Although at 5.2 years old, a dog can be considered within the first half of his life, there are studies reporting lower mean ages in both FRE and/or IBD dogs [23,24,25,26,27,28,29]. For complete information, there is also evidence of similar [30] or higher [31] mean age in FRE dogs, but the difference between the caseload of the present study and previous ones should be considered.

As expected, although evaluated over a relatively short period, while improving the clinical condition, the BCS passed from a value of 4.5 ± 0.6 at T0 to 5 ± 0.6 at FT. Interestingly, the BCS highlighted in the present cohort of dogs appears roughly similar to what was previously reported in the literature [22,31].

From a clinical point of view, both the indexes used in the study, the FS and the CCECAI, significantly improved from F0 to FT, respectively passing from median values of 6.0 and 5.0 to median values of 3.04 and 1.75; data were associated in all cases with the disappearance of diarrhea. The percentage of patients presenting with a normal FS passed from 19% (43/222) to 73% (163/222). In parallel, CCECAI was normal in only 28% of dogs included in the study at T0 (these dogs were not presenting normal stools) but passed to a normal value in 84% of patients at FT. As stated, not all patients had a normal fecal score or a CCECAI score less than or equal to 3 at FT. However, all patients were diagnosed with FRE because this disease can be diagnosed when clinical signs resolve or significantly improve after an appropriate dietary trial [2]. For FS, all dogs improved, and no patient had a score equal to or higher than 5 at FT; the same happened with the CCECAI, for which all scores improved, and none were found to be higher than 12 (value detected in only one dog), considering the inclusion in such an index of variables not directly related to the GI tract such as lethargy and dermatological signs. Furthermore, the median of the CCECAI passing from 5.0 to 1.75 suggested a general resolution of the condition across all subjects.

Regarding the specific clinical signs highlighted in patients enrolled, diarrhea was one of the most frequent ones, as witnessed by the high median FS, as previously reported in dogs suffering from adverse food reactions [21]. Also, vomiting was a highly recurrent sign in our patients, according to a previous study in which vomiting, even without diarrhea, was significantly associated in dogs with CE with FRE [32]. In the international literature, not many data are available about the monitoring of the clinical progression in dogs suffering from FRD and, in any case, neither considering the same variables (BCS, FS, CCECAI, hematochezia/melena, constipation, vomiting/regurgitation/signs of gastro-esophageal reflux disorder—GERD, weight loss, anorexia/dysorexia, lethargy, and dermatopathological signs, at T0 and control) nor in such a large cohort of patients as reported in the present study; therefore, although it is not possible to carry out particular comparisons with the existing literature, we believe that, as previously mentioned, these data can be useful as a term of comparison for future studies.

The multiple linear regression analysis highlighted what was expected, particularly in the relationship between BCS and FS to FT (inversely proportional) and CCECAI and FS to FT (directly proportional). On the other hand, interestingly, no significant correlation was found for almost any dietary shifts when compared to CCECAI at FT, with the only exception being the passage from mixed to homemade diet, which was significantly associated with an improvement in CCECAI.

As always regarding diet, it should be underlined that in the present study, considering its retrospective design, owners were free to decide the type of diet to be administered based on their preferences as a first- or second-line approach; therefore, not all patients underwent the same dietary trial, neither as the choice between the three options (commercial, homemade, and mixed diet), nor as the order of use. This aspect, which may be perceived as a weakness of the study, should also be seen in the light that we did not aim at assessing which dietary regimen could be the best one in resolving the clinical presentation. The fact that among the three options, the homemade diet proved to be the most successful one and the first owners’ choice in most cases only allows us to say that in our study, it was the one preferred by owners in such conditions and that it transpired to be effective in totally 107 cases (out of 222), without other possible comparisons among effectiveness of different dietary regimens. Interestingly, other authors prefer using a commercial hydrolyzed protein as a primary approach [2]. Recently, an amino acid-based kibble (providing protein as amino acids, a so-called “elemental diet”) was studied in dogs with inadequately controlled chronic enteropathy, and remarkably, it resulted in improved clinical signs in around 70% of patients, also modifying the GI microbiome in responders, opening up a possible new approach [29]. Another fascinating and actual theme is the evaluation of the possible associations, in dogs and cats suffering from FRE, between determined dietary regimens administered before the disease and the prevalence of the disease itself, but no ingredients are actually unanimously recognized as risk factors [4]. In a recent study on pre-illness dietary regimens in dogs with chronic enteropathy vs. controls, no carbohydrate diet was associated with controls, while few diseased dogs were fed with red meat as a primary protein source compared to controls, but the study of this matter needs and deserves to be further deepened [8]. Likewise, another study suggested that feeding dogs with a non-processed meat-based diet plus human meal leftovers during puppyhood/adolescence was protective against chronic enteropathy in adulthood, while feeding an ultra-processed carbohydrate-based diet during puppyhood/adolescence was a significant risk factor for chronic enteropathy later in life [33]. In this regard, in addition to the one previously reported, another possible limit of the study is that, unfortunately, we have only partial data about the dietary history of patients included in the study, and we were not able to accurately investigate which antigen they were exposed to before their inclusion, and for how long; this was however beyond the aim of the study.

Finally, another main aim of the study was to assess the possible association between breeds (divided into three subgroups based on the hypothetical expression of the *Amy2b* gene [16]), diet, and clinical progression, but unfortunately, no correlation was found, with the only exception of the passage from commercial to mixed diet in “red” breeds. Indeed, it was assumed that breeds included in the “red” subset, expressing low levels of the *Amy2b* gene, could show a better clinical response if fed a home or mixed dietary trial due to the lower starch content present in those dietary regimens, and our results are partially in agreement with this supposition. This hypothesis was substantiated by several published and anecdotal reports that describe a CIE predisposition in Nordic breed dogs [11] and Czechoslovakian wolfdog (which express low *Amy2b* levels) and their positive clinical response to a homemade elimination trial [34]. These authors, for instance, reported that representatives of Czechoslovakian wolfdogs with digestive disorders were free of gastrointestinal symptoms after being passed on an entirely grain-free diet. The partial correlation seen in our study between clinical response to diet and “red” subset breed may be explained considering that not only the starch-inclusion rate but also other factors, such as starch form (i.e., whole, broken, ground grains), sources (i.e., purified or flour starches), particle dimension, interaction with other nutrients, and structural changes due to technological processes, may contribute to carbohydrate tolerability [35]. These covariables, however, were not considered during our trial. Moreover, the low *Amy2b* gene encoding was hypothetically postulated and not precisely determined in breed subsets, leading to a possible misleading in breed categorization. Finally, the small cohort of patients in the “red” subcategory may have reduced the statistical power of the tests to underline a possible correlation between breed and diet.

## 5. Conclusions

The present study reports the clinical progression in 222 dogs suffering from FRE, and it could represent a reference for the variables investigated, considering the number of patients included. The diet chosen as a first-line approach by our patients’ owners was the homemade diet in 105 cases, and the shift from mixed to homemade diet was the only one significantly associated with an improvement in the CCECAI score. However, the study’s experimental design (retrospective) does not allow us to state the prevalence of one diet over the other. The hypothesis that patients involved in the study could have reacted to the dietary change in accordance with the hypothetical expression of the *Amy2b* gene, depending on their breeds, has not been confirmed (considering a single exception) but deserves further investigation through genetic evaluation.

## Figures and Tables

**Table 1:** In red are the breeds that hypothetically express a few copies of *Amy2b* (tot. n. 25 dogs), in green are those that express a high number of copies of the gene (tot. n 141 dogs), and finally, in yellow, the intermediate breeds between the two previous ones (tot. n 56 dogs). In brackets, the number of animals relating to each breed.

Akita inu (7)	Boxer (19)	Mixed breed (45)
Czechoslovakian wolfdog (6)	Weimaraner (9)	French bulldog (13)
Afghan hound (2)	Chihuahua (6)	German shepherd (10)
Dachshund (2)	Cavalier King Charles spaniel (4)	Golden retriever (9)
Alaskan malamute (1)	English setter (4)	Poodle (9)
Chow chow (1)	German shorthaired pointer (2)	American Staffordshire terrier (7)
Maremma sheepdog (1)	West Highland white terrier (2)	Labrador retriever (7)
Pug (1)	Yorkshire terrier (2)	Jack Russell terrier (5)
Saluki (1)	Brittany spaniel (1)	Miniature Pinscher (4)
Shiba inu (1)	Irish setter (1)	Pomeranian (4)
Shih-tzu (1)	Italian greyhound (1)	Bernese mountain dog (3)
Whippet (1)	Italian hound (1)	English bulldog (3)
	Italian pointer (1)	Maltese (3)
	Lagotto (1)	Pittbull (3)
	Rhodesian ridgeback (1)	Rottweiler (3)
	Vizsla (1)	Beagle (2)
		Cocker spaniel (2)
		Argentine dogo (1)
		Australian cattle dog (1)
		Australian shepherd (1)
		Bolognese (1)
		Border collie (1)
		Corso (1)
		Doberman (1)
		Flat-coated retriever (1)
		Staffordshire bull terrier (1)

**Table 2:** Values at T0 and FT of selected clinical parameters: body condition score (BCS), fecal score (FS), and canine chronic enteropathy activity index (CCECAI). In bold, significant *p* values.

	T0	FT	*p* Values
Body weight (mean ± DS)	22.1 kg ± 11.3	22.73 ± 10.9	*p* = 0.403
BCS (median ± DS)	4.5 ± 0.6 (from 1 to 9)	5.0 ± 0.6	***p* < 0.001**
FS (median ± DS)	6.0 ± 1.6 (from 1 to 7)	3.04 ± 1.2	***p* < 0.001**
CCECAI (median ± DS)	5.0 ± 4.0 (from 0 to 27)	1.75 ± 2.2	***p* < 0.001**

**Table 3:** Frequency of selected clinical variables/signs evaluated at T0 and FT. In brackets and bold, the number of animals (out of 222) and related percentages presenting the clinical sign. * *p* < 0.05; *** *p* < 0.001.

Frequency of Clinical Signs at T0 (n—%)	Frequency of Clinical Signs at FT (n—%)
hematochezia/melena **(21–9.46)**	hematochezia/melena (6–2.70) ***
constipation **(1–0.45)**	constipation (0–0) *
vomiting/regurgitation/signs of gastro-esophageal reflux disorder (GERD) **(100–45.04)**	vomiting/regurgitation/signs of gastro-esophageal reflux disorder (GERD) (56–25.22) ***
weight loss **(71–31.98)**	weight loss (33–14.86) ***
anorexia/dysorexia **(35–15.77)**	anorexia/dysorexia (14–6.31) ***
lethargy **(22–9.91)**	lethargy (6–2.70) ***
dermatopathological signs: alopecia, pruritus, otitis **(55–24.77)**	dermatopathological signs: alopecia, pruritus, otitis (49–22.07) ***

**Table 4:** Multiple linear regression analysis between covariables. In bold, significant *p* values.

VARIABLES	FS (FT)
Age	***p* < 0.01** (r = −0.1908)
Body weight	*p* = 0.4615 (r = 0.04967)
BCS (FT)	***p* < 0.01** (r = −0.2186)
CCECAI (FT)	***p* < 0.001** (r = 0.2308)
**DIETARY SHIFT**	**CCECAI (FT)**
From commercial to homemade diet	*p* = 0.3304 (r = 0.06562)
From commercial to mixed diet	*p* = 0.8720 (r = 0.01088)
From homemade to commercial diet	*p* = 0.6110 (r = 0.03432)
From homemade to mixed diet	*p* = 0.2467 (r = −0.07806)
From mixed to commercial diet	*p* = 0.8225 (r = 0.01514)
From mixed to homemade diet	***p* < 0.05** (r = −0.1398)

**Table 5:** Dietary variations regarding first and second (where necessary) successful options, based on owners’ preferences.

	Commercial Diet	Homemade Diet	Mixed Diet
		(n of Patients)	
First option (successful)	56	105	58
Second option (successful)	0	2	1
**Tot. 222**	**56**	**107**	**59**

## Data Availability

The original contributions presented in the study are included in the article, further inquiries can be directed to the corresponding author.
